# Supplementary material for: Increased Serum IgG4 Associates with Asthma and Tissue Eosinophilia in Chronic Rhinosinusitis Patients
Source: Pathogens. 2020 Oct 10;9(10):828. doi: 10.3390/pathogens9100828 (PMC7600683; doi:10.3390/pathogens9100828)
Supplement: Supplementary file 1 [file pathogens-09-00828-s001.pdf]

Methods

Patients and Samples

This study was approved by the Human Research Ethics Committee of The Queen Elizabeth Hospital, Adelaide, Australia (reference number: HREC/15/TQEH/132). Biopsies were obtained from the ethmoid sinuses from patients undergoing endoscopic sinus surgery at the Department of Otolaryngology, Head and Neck Surgery, The Queen Elizabeth Hospital, Adelaide, Australia. Control patients were patients undergoing endoscopic skull base procedures without clinical or radiological evidence of sinus disease. CRS patients fulfilled the diagnostic criteria set out in the recent position papers by the American Academy of Otolaryngology and Head and Neck Surgery and the European Position Statement (EPOS) on CRS [1,2]. Patients with CRS were further sub-classified according the absence (CRSsNP) or presence (CRSwNP) of visible polyps present within the middle meatus on nasal endoscopy as defined by EPOS guidelines and both polyp and mucosal tissue was taken for CRSwNP patients [1,2]. Serum samples were obtained from CRSsNP, CRSwNP and control patients.

Preparation of Protein Extract from Tissues

Freshly obtained nasal polyps and mucosal tissues were snap-frozen and stored at −80 °C until needed. Tissues were thawed on ice. Approximately 100 mg of tissue was suspended in 1 mL of T-per tissue protein extraction buffer (product no. 78510, Thermo Fisher Scientific, MA, USA) containing 1% v/v HALT protease inhibitor cocktail (product no. 87786, Thermo Fisher Scientific, MA, US). The samples were then homogenized with Qiagen Tissuelyser (product no. 85220, Qiagen, Hilden, Germany) at 30 hertz for 20 min. The homogenized suspensions were centrifuged at 17,000 g for 10 min at 40 °C and the supernatants were stored at −80 °C until analysis. The protein concentrations of tissue extracts were determined using the Pierce BCA Protein Assay Kit (product no. 23225, Thermo Fisher Scientific, MA, USA).

Measuring IgE, Total IgG, and IgG Subset (IgG1, IgG2, IgG3, IgG4) Levels in Serum Samples

IgE, total IgG, IgG subsets (IgG1, IgG2, IgG3, IgG4) measurements in serum samples were carried out using the ImmunoCAP ® 1000 System (Clinpath, SA, Australia).

Histology

Paraffin-embedded tissue samples were cut in 4μm thickness, stained with Haematoxylin & Eosin (H&E) and scanned using digital whole-slide imaging (WSI) technology (NanoZoomer, Hamamatsu, Japan). Tissue eosinophilia was determined by averaging the number of eosinophils per High Power Field (HPF) (0.035 mm^3) from at least 6 HPF's/slide as specified by Ramezanpour et al [3].

Immunohistochemistry Analysis

Slides were deparaffinized and rehydrated. Antigen retrieval was induced at 100 ºC for 10 min in 10 mmol/L sodium citrate buffer, pH 6. Slides were then blocked in 25% normal horse serum blocking buffer for 10 min and incubated with primary antibodies to IgG4 (mouse antihuman IgG4 [A10651] at 1:200 dilution; life technology, OR, USA) or to IgE (rabbit monoclonal [ab195580] at1μg/ml; Abcam, Cambridge, United Kingdom) overnight at 4 ºC.

Specific binding was detected with the Vectastain Universal Quick kit #PK-7800 and DAB Substrate kit #SK-4100 (Vector Laboratories, Burlingame, CA, USA). Slides were observed with a Nikon Eclipse 90I microscope equipped with NIS-Elements AR3.2 software.

Detection of Autoantibodies

The presence of autoantibodies in serum was tested against 12 Anti-Nuclear Antigen (ANA)-associated autoantigens (nRNP, Sm, SS-A (SS-A native and Ro-52), SS-B, Scl-70, Jo-1, CENP B, dsDNA, Nucleosomes, Histones and ribosomal P-Protein) were tested using Euroimmun ANA Profile I Immunoblot kits (catalogue no. DL 1590-6401-8G, Euroimmun, Lübeck, Germany). The tests were performed as per the manufacturer's manual. The blots were evaluated by the EUROLineScan (Euroimmun, Lübeck, Germany) for scanning and analysis. Background cut-off value was ≤ 10 intensity unit. 3 serum samples known to possess ANAs were used as positive controls.

Statistical Analysis and Principal Component Factor Analysis of Serum Immunoglobulin

Principal component factor analysis was performed to both interpret the relationships between serum immunoglobulins and to simplify the analysis of between-group differences. With the exception of total IgG, none of the immunoglobulin measurements were normally distributed, therefore, normalising transformations were applied based on the Tukey ladder of powers. Log transformations were selected for IgE and IgG4, and square root transformations were selected for IgG1, IgG2 and IgG3. Transformed variables were then standardised before principal component factor analysis. Analysis was performed in Stat v15.1 (StataCorp LLC, TX, USA).

Table S1. Principal components factor analysis of serum immunoglobulin levels showing varimax rotated factor loadings for the three identified factors. Factor 1 captures total IgG, and the most abundant subtypes IgG1 and IgG2, Factor 2 captures IgG4 and IgE and Factor 3 captures IgG3.

Variable	Factor1	Factor2	Factor3
IgE loading	0.00	**0.91**	0.16
IgG loading	**0.97**	0.18	0.11
IgG1 loading	**0.83**	-0.20	0.19
IgG2 loading	**0.76**	0.30	0.10
IgG3 loading	0.28	0.10	**0.90**
IgG4 loading	0.47	**0.62**	-0.45
Eigenvalue	2.81	1.28	0.91
%Variance explained	46.9	21.3	15.1
%Cumulative variance	46.9	68.2	83.3

Table S2. Median (interquartile range) of serum immunoglobulin IgG subtype and IgE levels in controls, chronic rhinosinusitis patients without nasal polyps (CRSsNP) and chronic rhinosinusitis patients with nasal polyps (CRSwNP). *p*-values were derived from an exact test for differences in the medians between the three groups.

Immunoglobulin	Control	CRSsNP	CRSwNP	*p*
	n = 10	n = 10	n = 26	
IgG (g/L)	8.56 (3.06)	12.35 (5.02)	11.65 (2.63)	0.001
IgG1 (g/L)	5.65 (2.45)	7.88 (3.05)	6.44 (2.38)	0.018
IgG2 (g/L)	2.07 (0.74)	3.36 (1.92)	3.96 (2.22)	0.010
IgG3 (g/L)	0.27 (0.26)	0.36 (0.22)	0.28 (0.25)	0.92
IgG4 (g/L)	0.26 (0.19)	0.37 (0.29)	0.87 (0.91)	0.002
IgE (IU/mL)	26 (40)	78.5 (194)	141.5 (246)	0.010

References

1. Fokkens WJ, Lund VJ, Mullol J, Bachert C, Alobid I, Baroody F, et al. European Position Paper on Rhinosinusitis and Nasal Polyps 2012. Rhinology Supplement. 2012(23):3 p preceding table of contents, 1-298.

2. Lin SY, Nnacheta LC. American Academy of Otolaryngology-Head and Neck Surgery Foundation (AAO-HNSF) will publish its latest "Clinical practice guideline: allergic rhinitis (AR) in February, 2015". American journal of rhinology & allergy. 2015;29(1):82.
3. Ramezanpour M, Moraitis S, Smith JLP, Wormald PJ, Vreugde S. Th17 Cytokines Disrupt the Airway Mucosal Barrier in Chronic Rhinosinusitis. Mediators of inflammation. 2016;2016:7.
